# Evaluating the Acceptability, Feasibility, and Outcomes of Two Methods Involving Patients With Disability in Developing Clinical Guidelines: Crossover Pilot Study

**DOI:** 10.2196/24319

**Published:** 2021-11-23

**Authors:** Marie-Eve Lamontagne, Marie-Pierre Gagnon, Kadija Perreault, Véronique Gauthier

**Affiliations:** 1 Faculté de médecine Université Laval Québec, QC Canada; 2 Centre interdisciplinaire de recherche en réadaptation et en intégration sociale Québec, QC Canada; 3 Faculté des Sciences infirmières Université Laval Québec, QC Canada; 4 École de travail social et de criminologie Faculté des sciences sociales Université Laval Québec, QC Canada

**Keywords:** clinical practice guidelines, patient participation, traumatic brain injuries, clinical practice, patient, participation, brain injury, traumatic

## Abstract

**Background:**

Engaging patients and the public in clinical practice guideline (CPG) development is believed to contribute significantly to guideline quality, but the advantages of the various co-design strategies have not been empirically compared, making it difficult to choose one strategy over another.

**Objective:**

This pilot study aims to document the acceptability, feasibility, and outcomes of 2 methods of involving patients in outlining CPG.

**Methods:**

A single-blind crossover pragmatic study was performed with patients with traumatic brain injury. The patients experimented with 2 alternative methods of producing clinical practice recommendations (ie, a discussion group and a wiki). The participants rated the acceptability of the 2 methods, and feasibility was assessed using indicators, such as the number of participants who completed the 2 methods and the number of support interventions required. Experts, blinded to the method, independently rated the participants' outcome recommendations for clarity, accuracy, appropriateness, and usefulness.

**Results:**

We recruited 20 participants, and 16 completed the study. The acceptability of the 2 methods showed little variation, with qualitative comments expressing a slight preference for the social nature of focus groups. Thus, both methods of involving patients in CPG development appeared feasible, and the experts’ opinions of the adapted recommendations were both positive, although the recommendations produced through focus groups were deemed more relevant to support clinical practice.

**Conclusions:**

Our results confirm the acceptability and feasibility of focus groups and wikis to allow patients with traumatic brain injury to participate in clinical practice guideline production. This study contributes to the scientific literature by suggesting that the 2 methods were acceptable, feasible, and produced positive outcomes.

**Trial Registration:**

ClinicalTrials.gov NCT02023138; https://clinicaltrials.gov/ct2/show/NCT02023138

## Introduction

Patient and public involvement (PPI) in the development of clinical practice guidelines (CPG) can contribute significantly to their overall quality [1]. The Guideline International Network published an updated toolkit on patient and public involvement in guidelines [2], and many other organizations, such as the United States’ Institute of Medicine [3] and United Kingdom’s National Institute for Clinical Excellence [3], recommend such patient and public involvement [4]. In the general field of medicine, Légaré et al [5] describes three broad types of PPI in CPG: (1) communication of the CPG to the target patient population, (2) consultation with the patient population through paper or online surveys, focus groups, or interviews, (3) direct participation of patients and public in CPG development, using direct involvement of these individuals in consensus meetings and development groups. Despite their increasing legitimacy and use, little is known about the best methods to engage patients and the general public in CPG production [6,7]. This lack of knowledge can compromise PPI in CPG development [1]. It may even impact patient and provider adherence with the CPG and ultimately the quality of care provided.

As synthesized in a recent scoping review by Kim et al [7], a large number of methods of PPI in CPG production are reported in the literature, combined or not, such as surveys [8], wikis, focus groups [9], in-depth interviews [10], online Delphi methods [11,12], and workshops [13]. PPI experiences are associated with a range of challenges, described by Armstrong et al [14] as a lack of clarity about the roles and tasks of patients in the CPG development process; limited resources for supporting patients; integrating service user input with supporting evidence from the literature in the CPG; ensuring that participating service users represent the views of service users as a group; the ability of service users to voice their concerns in the context of CPG development; recruitment difficulties; representativeness of selected participants; training and support needs of patient and population; patient representatives’ feelings of isolation; and difficulty with medical (or scientific) terminology, and systematic review process [4,14]. In addition, van de Bovenkamp et al [6] believe that the challenges faced by patients in the CPG development process highlight possible inconsistencies between the context, the strategy used, and the needs and capacities of patients. However, as reported by Kim et al [7], the above can be mitigated through training in research or CPG development methods and combining multiple methods. The World Health Organization recommends stakeholder involvement insofar as it is “feasible and efficient” [2,15] while recognizing that PPI strategies are not all equivalent, and the characteristics specific to each influence their feasibility and effectiveness.

Few studies have used formal study designs to empirically investigate strategies involving patients and the public in CPG development, whether from the perspective of the process (ie, acceptability and feasibility) or effectiveness. As part of the North of England evidence-based guideline development program, Van Wersch and Eccles [16] tested the participation of individual patients with asthma or angina in multidisciplinary CPG development groups through one-off meetings or workshops and a consumer advocate. The authors concluded that no method stood out as superior to the others and that further work was required on the best way to get meaningful PPI in CPG development [16]. Diaz Del Campo et al [17] reported their experience of combined methods of PPI using in-depth interviews, focus groups, and active participation of patients in all steps of CPG development for anxiety, insomnia, autism, and stroke within the Spanish National Clinical Practice Guideline Development Program. They found that PPI in CPG development was very helpful in incorporating patient views and needs in the guidelines, but it was crucial to provide specific support to patients to facilitate effective engagement [17]. Khodyakov et al [12] recruited 95 participants to evaluate the patient-centeredness of CPG for people with Duchenne muscular dystrophy using an online modified Delphi method. Up to 56% of their sample participated in all three rounds of discussions, and the authors observed that this method allowed them to consider the patient-centered nature of the guideline recommendations effectively. Serrano-Aguilar et al [18] used Delphi-based consultations combined with a systematic review to include patient perspectives into CPG for persons living with systemic lupus erythematosus. They concluded that the method was useful to align clinical practice with users’ needs better. Köpke and al [8] used a survey and focus groups to consult individuals with severe multiple sclerosis about palliative care. Despite the time and resource-intensive character of the activity, user input was deemed key in formulating the guideline questions and identifying outcomes. Den Breejen et al [19] evaluated an approach to the simultaneous development of 5 guidelines on infertility. They used exploratory interviews with 12 couples facing fertility issues, 2 focus groups, and a wiki tool over 7 months. Among other results, the authors concluded that the wiki was a promising and user-friendly tool for patient participation in guideline development and for identifying targets for CPG improvement.

Although important insights have recently been gained in understanding PPI in CPG development, to our knowledge, no experimental study has compared 2 methods of PPI concerning their acceptability, feasibility, and outcome. The absence of comparative data makes it difficult for guideline developers to choose one method over another. In addition, despite the wide range of patient populations that have contributed to CPG production, to our knowledge, individuals with cognitive disabilities, such as those induced by traumatic brain injuries (TBIs), have never been involved. Co-design of products and interventions for this patient population is recent [20-22] and has been described as challenging but fruitful, as special attention has to be given to adapting the methods of involvement to the capacities and limits of these individuals to ensure their feasibility. For example, the number of individuals with TBIs that can be involved at the same time in a focus group is limited. Furthermore, clear and enhanced instructions must be provided and restated regularly to ensure that co-design can occur. The goal of this pilot study was to document the acceptability, feasibility, and outcome of 2 methods of PPI in CPG co-design for adults with TBIs.

## Methods

### Study Design

We performed a single-blind crossover pragmatic study [23,24] with patients with TBIs in the context of a rehabilitation guideline development. TBI is defined by the Center for Disease Control [25] as a disruption in the brain's normal function that can be caused by a bump, blow, or jolt to the head or a penetrating head injury. It leads to various and often permanent physical and cognitive sequelae such as fatigue, trouble concentrating, and memory loss [26,27]. The crossover pragmatic pilot study design allows participants with this condition to act as their own control while enabling minimum control for both learning and fatigue between the 2 methods. The complete protocol of the study was published elsewhere [28]. The patients participated in 2 alternative co-design methods of recommendations (ie, a focus group and a wiki). Patients were asked to discuss 2 recommendations chosen from the Scottish Intercollegiate Guideline Network guidelines for the rehabilitation of individuals with TBI [29]. This guideline was assessed as having a high-quality score on the AGREE II tool [30]. Because individuals with TBI may have various clinical profiles and trajectories, we chose recommendations that are relevant for a wide range of participants to facilitate their involvement. The recommendations to be adapted were selected by 2 researchers (MEL and MPG)

**Recommendation 1**: For optimal outcomes, higher intensity rehabilitation featuring early intervention should be delivered by specialist multidisciplinary teams. (Scottish Intercollegiate Guideline Network: Brain injury rehabilitation in adults*.* Edinburgh: SIGN; 2013. p. 38)

**Recommendation 2**: Planned discharge from inpatient rehabilitation to home for patients who have experienced an acquired brain injury provides beneficial outcomes and should be an integrated part of a treatment program. (Scottish Intercollegiate Guideline Network: Brain injury rehabilitation in adults. Edinburgh: SIGN; 2013. p. 43)

The research protocol was approved by the Research Ethics Committee of the Institut de réadaptation en déficience physique de Québec (Québec Rehabilitation Institute for Physical Disability 2013-0348). Our pilot study is reported using PRISMA (preferred reporting items for systematic reviews and meta-analyses) extension for pilot trial criteria.

### Participants

A convenience sample of patients meeting the following criteria was selected: (1) having suffered a moderate to severe TBI (Glasgow Coma Scale <13) between 2 and 4 years previously to ensure they still remember their completed rehabilitation process, (2) being French-speaking, (3) being able to use a computer, and (4) having the physical and cognitive capabilities to participate in a 2-hour group meeting. Participants were recruited among members of the Quebec community-based association of individuals with TBI (Association TCC des Deux-Rives). These participants were chosen because this association regroups many individuals with TBI from rural and urban settings and those who live close enough to participate in an in-person focus group at the association. A letter containing information about the project was sent to a random list of 30 members of the association who met the eligibility criteria and were deemed able to participate in the project by an association employee. One week later, the association employee contacted members by phone using a recruitment script to answer any questions and verify their interest in participating in the study. The final list of 20 (66.6%) interested members was forwarded to the researchers.

A research assistant contacted the potential participants by phone to confirm their eligibility and obtain consent. The participants were invited to attend a training session that was to be held one week later.

### Procedures

**Figure 1:**
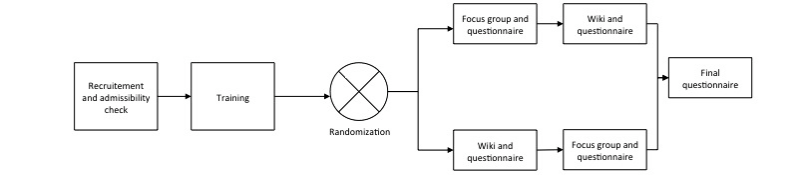
Study design and concept measurement.

The study compared 2 methods of PPI (ie, focus group and wiki). The participants tried each method in accordance with the crossover design (Figure 1). The study activities took place at the association’s office (for the training session and focus group) and the participant’s home (wiki).

### Training

The participants received in-person training about the guidelines and PPI using educational material on the subject developed by the Health Council of Canada [31]. Training was essential to ensure that participants had some knowledge about CPG and PPI and could participate effectively in the study. The participants were also given information about the 2 methods to be tested and a written process outline to support their participation in the study and accommodate potential cognitive problems. Each participant also answered a sociodemographic questionnaire covering self-reported trauma characteristics. The training meeting lasted one hour to avoid fatigue.

After the training, participants were randomized into Group 1 or Group 2 by a researcher blinded to the intervention, using a random number generator [32]. The allocation concealment was performed using the list of participants randomly ordered.

### Focus Group

Group 1 participants were first invited to participate in a 2-hour focus group aimed at co-designing the particular recommendation to encompass their values and preferences. Three days before the focus group, they were emailed: (1) instructions about the time and place of the group meeting, (2) the recommendation to be discussed, and (3) a synthesis of the scientific articles that were used to produce that specific recommendation. The focus group was moderated by the principal investigator (MEL). It began with a quick reminder about the goal of the study and the goal of the focus group. The synthesis of the evidence used to produce the recommendation was reviewed, and the participants were asked to discuss the recommendation. The objective of the discussion was to get the participants’ opinions about the recommendation, elicit the participants’ preferences about the recommendation, and explore possible changes to be made to the recommendation. The results of the discussion were recorded on flip charts as the discussion proceeded. These visual cues facilitated the participation of users who have cognitive problems. The focus group was also audiotaped, and its content transcribed for qualitative analysis.

### Wiki

Group 2 participants were invited to participate in a wiki. A wiki is a collaborative writing web application used to create online content that anyone can edit or add to [33,34]. The wiki was chosen as an innovative intervention that would allow participants to be involved in the CPG co-design in a place and at a pace best suited to their abilities as persons living with TBI and without having to overcome transportation barriers.

Group 2 participants received an invitation by email, including clear and detailed instructions about wiki use, the recommendation to be discussed online (the same one used by Group 1), and a synthesis of the evidence used to produce that specific recommendation. The wiki was structured to describe the research project, present a synthesis of the evidence used to produce that specific recommendation, allow the participants to express their opinions concerning the recommendation, specify their preferences about the recommendation, and suggest potential modifications to the recommendation. The participants also received prompts from the researchers 2, 4, and 6 days after the initial email to encourage them to participate in the wiki discussion. They had 1 week to do so.

One week after the end of the first data collection, in accordance with the crossover design, Group 1 participants were assigned to the wiki method, and Group 2 participants were assigned to the focus group method. The procedure was repeated with a second recommendation different from the first.

### Instruments and Measures

#### Acceptability

We assessed the acceptability of both methods for individuals with TBI after using each method, using electronic surveys for the wiki and paper surveys for the focus group. We developed the questionnaire using Sidani and Braden’s conceptual framework of acceptability [35]. In line with this framework, the questionnaire measured the participants’ perceptions with regard to the appropriateness of the method identified, fit with usual habits, perceived effectiveness, perceived consequences, and the likelihood of re-using the method. For each aspect, the participants were asked to rate their agreement with a statement using a 10-point visual analog scale ranging from “0” (I totally disagree with this sentence) to “10” (I totally agree with this sentence). They were also invited to explain their answer using an open-ended qualitative question. After testing both methods, the participants were also asked to answer a final questionnaire to validate their preferences regarding the 2 methods. The questionnaires were pretested with 3 individuals with TBI who would have been eligible but did not participate in the study to ensure language clarity and questionnaire comprehensiveness.

#### Feasibility

The feasibility of the intervention was evaluated using 3 indicators: (1) the number of participants who attended the focus group or accessed the wiki, (2) the number of participants who completed the intervention (ie, attended the entire duration of the focus group or provided their opinion on the recommendations), and (3) the number of support interventions required regarding the focus group and the wiki (eg, a telephone call for assistance, email, or request to the association’s employees) [35]. The indicators were documented by a research professional, using an Excel (Version 16.13.1 /2018; Microsoft Corporation) preformatted template form.

#### Outcomes

The outcomes of the 2 methods were evaluated by submitting the adapted recommendations to a panel of potential users, including individuals with TBI, clinicians, managers, and policymakers. They were recruited from the scientific committee overseeing a TBI guideline adaptation process and from the community-based association. In this study, co-design outcomes were understood as the capacity of a method to produce recommendations that are clear, appropriate, and useful in guiding users in their activities. The experts were blinded to the methods used to adapt the recommendations. They received an email including the original recommendations, the recommendations co-designed by the participants, and a link to a questionnaire in which they were invited to independently rate the clarity, accuracy, appropriateness, and usefulness of each of the 4 recommendations (2 methods x 2 recommendations) for their practice using 10-point Likert scales. They were also asked to provide comments to justify their ratings.

#### Analysis

Within-subject analyses using the Wilcoxon signed-rank test [36] were performed to compare the acceptability score of the 2 methods. Feasibility indicators were reported using descriptive statistics. The outcomes of the 2 methods as rated by the experts were compared using generalized linear models (2 methods x 2 recommendations, generalized estimating equations, and both independent variables repeated measures). The statistical analyses were performed using SPSS (version 20; IBM Corp). All qualitative data (focus group and qualitative comments stemming from the questionnaires) underwent a thematic content analysis [37-39] based on Sidani and Braden’s conceptual framework of acceptability [35] using Nvivo software (version 12; QSR International). An interjudge validation process was done by 2 team members to ensure the validity of the analysis until a level of agreement of 90% was found in the coding. Divergences in coding were resolved by a third analyst (MPG). Finally, themes were validated according to convergences and divergences.

## Results

### Participants

In total, 20 participants with TBI were recruited to participate in the study; 1 individual dropped out during the training (not interested), 3 left before the randomization (2 because of lack of time and 1 because of sickness), and the remaining 16 participants completed the study. Characteristics of the participants are listed in Table 1. Most of the injured participants were male; their median age was 48 years, and they had experienced a severe TBI more than 15 years before. Half of the participants lived alone. None of the participants had previous knowledge of CPG. Both groups were similar in terms of sociodemographic characteristics.

We recruited 18 experts (5 clinicians, 5 managers, 4 decision-makers, and 4 individuals with TBIs who were potential users of the CPG); 11 (61%) were female, 9 (50%) were aged between 45 and 64 years, 5 (28%) between 35 and 44 years, and the remaining 4 (22%) participants were under 34 years old. Five (28%) experts had a Ph.D. as their highest level of education completed, 4 (22%) had a Master’s degree, 5 (28%) a Bachelor’s degree, and the remaining 4 (22%) participants had a college or professional diploma. Experts rated their knowledge of guidelines at 64% on average and use at only 42% (on a scale of 0 to 100%).

**Table 1:** Sociodemographic characteristics of the participants living with a traumatic brain injury.

		Traumatic brain injury participants		
		Group 1 (n=8)	Group 2 (n=8)	All (n=16)
Male, n (%)		5 (62.5)	6 (75%)	11 (68.8)
Age (years), median (range)		45 (20-57)	44.4 (26-57)	44.9 (20-57)
Severe traumatic brain injury, n (%)		5 (62.5)	5 (62.5)	10 (62.5)
**Living situation,** **n (%)**				
	Alone	5 (62.5)	4 (50)	9 (56.3)
	With close relatives	3 (32.5)	4 (50)	7 (43.4)
Knowledge of CPG existence, n (%)		0 (0)	0 (0)	0 (0)

### Acceptability

The participants’ rating of acceptability of the 2 methods (Table 2) did not differ significantly *(P* values varying from .12 for “methods had some advantages” to .69 for “methods is simple”). Overall, both methods received higher scores for simplicity, likelihood to be used again and appropriateness, and lower scores for convenience. The participants found that the methods, especially the focus group, had more advantages than disadvantages, although this was not statistically significant.

The qualitative comments offer a more nuanced view of the scores provided regarding method acceptability. Overall, the focus group method was perceived more positively by the participants. It was deemed to be simpler by participants because they only had to talk. Listening to other participants also facilitated reflection and contributed to the ease of use of this method:

All the comments made helped me to think about my own experience. And it helped me to see other aspects I hadn’t thought of before”

participant #8

The focus group was said to more easily fit with the communication habits of individuals with a head injury who prefer oral expression to writing. The focus group also allowed them to express their thoughts faster. However, participants recognized that it was sometimes difficult maintain concentration and comment on the recommendation in a noisy environment. The main perceived advantage of the focus group method was its social aspect, which met the needs of individuals with brain injury (often socially isolated). The main disadvantage of the method was that it was sometimes difficult for them, as individuals with cognitive disabilities who may experience memory and concentration issues, to participate in the discussion in a sustained manner. Participants also mentioned that on several occasions, they felt an obligation to go in the same direction as the group to reach a consensus about the recommendation, even though their personal experience was different from what others described:

We have to fit everybody’s ideas into one, and it is not always easy, especially when you have difficulty expressing yourself

participant #6

The focus group was viewed as appropriate and convenient by a majority of participants, allowing the moderator and participants to summarize a wide range of individual experiences into a few simple statements. Fatigue, fear of being judged, and difficult memories brought back through trauma recall were the most often cited drawbacks of the focus group method. Despite this, a vast majority of participants stated that they would use this method again because it is rewarding, enlightening, and has a positive social dimension.

Regarding the wiki method, the participants explained that it was simple to use thanks to the detailed instructions provided, but the required access to a computer and internet was a constraint. While some participants mentioned that they were accustomed to using electronic communication media (eg, Facebook and email), most said they would rather have a face-to-face conversation:

I prefer to discuss my ideas in person with real people, especially when I have questions

participant #8

Participants considered the wiki method effective to the extent that it allowed for analysis and reflection before answering and because participants could express themselves without being stopped or interrupted. Other key advantages mentioned included anonymity, not feeling judged by others, the freedom to choose the time and place to answer the wiki questions, and doing it at the participant’s own pace, respecting his or her capacity.

**Table 2:** Acceptability, feasibility, and outcomes of focus group and wiki.

		Focus group	Wiki	*P* value	
**Median and range of scores of acceptability items for participants with traumatic brain injuries (%), median (range)**					
	Simple	93 (8-100)	90 (1-100)	.69	
	Fit usual communication habits	90 (8-100)	70 (0-100)	.31	
	Effective	88 (8-100)	85 (0-100)	.58	
	Had some advantages	88 (8-100)	61 (0-100)	.11	
	Had some disadvantages	65 (0-100)	61 (0-100)	.48	
	Appropriate	92.5 (0-100)	88 (0-100)	.59	
	Convenient	84 (0-100)	65 (0-100)	.48	
	Would like to use this method again	97.5 (8-100)	91 (1-100)	.48	
**Feasibility**					
	Number of participants who used the method	16	16	—^a^	
	Number of dropouts	0	0	0	
	Number of support requests	0	1	0	
**Outcomes as perceived by the experts**					
	Mean clarity score of the recommendation	67% (1-93%)	57% (12-99%)	—	
	Mean precision score of the recommendation	68% (6-90%)	60% (42-100%)	—	
	Mean usefulness score of the recommendation	67% (24-95%)	60% (0-99%)	—	
Model effect of the method		Wilks Lambda value: 0.779			.08
Model effect of the recommendation		Wilks Lambda value: 0.875			.24

^a^Statistical test not preformed.

### Feasibility

Both methods appeared highly feasible as a way of involving patients in CPG co-design. After randomization, no participants dropped out from the focus group or the wiki intervention. All participants used both methods. In addition, the participants required little support; only 1 participant asked for help once to access the wiki platform (Table 2).

### Outcomes

The quantitative analysis of the experts’ opinions illustrates positive perceptions of experts for both methods. No significant differences were found between the 2 methods (Wilks Lambda=0.079; *P=*.07) and between the 2 recommendations (Wilks Lambda=0.0.85; *P=*.24). When asked whether they preferred the recommendations designed through the focus group or wiki, a majority of experts mentioned that the recommendations produced through the focus group appeared more relevant to their rehabilitation practice. This result was reinforced by the fact that means and median scores of clarity, appropriateness, and usefulness were systematically higher for the focus group, although differences were not statistically significant. Furthermore, the qualitative comments point out that the recommendations produced through the focus group were clearer and more accurate.

## Discussion

The objective of this pilot study was to evaluate 2 methods of involving patients in CPG co-design, a focus group and a wiki. Overall, we found that both methods were judged acceptable and feasible, and it allowed participants to contribute to co-design recommendations based on their values and experiences. While the data obtained via questionnaires did not show any difference between both methods in terms of acceptability and outcomes, qualitative comments indicate that focus groups may present more advantages than the wiki for individuals with TBIs.

Acceptability refers to the patient’s view of an intervention that strongly influences its uptake, implementation, and adherence to CPG [35]. Obtaining patient perspectives on the strategies they view as most appropriate is of crucial importance for effective and sustainable PPI in CPG design. In our study, the focus group and wiki methods were both acceptable, and they presented distinctive features (eg, social aspects, inspiring discussions for focus groups, no time constraint, geographically accessible, and easier concentration for the wiki method) that are susceptible to accommodate individuals with TBI who experience various disabilities. Our study shows that the little training required for focus group participation, the possibility to clarify the technical or scientific language used, and the positive feelings that participation can elicit in a social group might counter important support needs [40], such as patient representatives’ feelings of isolation [9], and difficulty with medical terminology [41,42] which have been identified as obstacles to PPI in the literature.

Although the wiki method can be considered cost-effective, especially in terms of remote use, it may fail to satisfy the social aspect of group participation provided by other methods. Furthermore, explaining terminology is easier in person than through a wiki. However, asynchronous and remote participation modes in CPG co-design remain important facilitators that allow a range of patient populations, such as individuals with important physical disabilities or impairments due to fatigability, to contribute to CPG design.

However, the feasibility of PPI in CPG design relies on factors such as resources required and the time and availability of participants. Based on our results—no dropout, high access, and little need for support observed—the feasibility of the focus group and wiki methods is high. One possible explanation is a positive social bias, where the contact with the research team generated positive feelings and commitment. Given that individuals living with TBIs often face social isolation, they may react more positively to solicitation than other populations. Another explanatory hypothesis for the high degree of feasibility associated with both methods lies in the training and support material provided to the participants. Our results support the argument highlighted in the literature that participants need assistance to participate in CPG design effectively, although this was provided before experimentation in this study and was barely required afterward. However, the commitment observed to CPG design may also reflect the importance of the topic for patients and their appreciation of being offered the opportunity to cast their voice in a matter of interest to them.

Bovenkamp et al [6] argue that the effectiveness of different methods of including PPI in CPG design is difficult, though not impossible, to evaluate using a randomized clinical trial design. Kim et al [7] found that few studies explicitly evaluate the processes or impact of identifying or incorporating patient preferences and fail to report the impacts of such involvement on CPG outcomes. The results of our pilot study suggest that the recommendations co-designed through focus group and wiki were both judged positively in terms of outcomes by a group of experts, including patients, providers, decision-makers, and researchers, with no statistically significant differences between methods. Comments they provided nonetheless indicate that the ones developed through focus groups were superior. Few researchers have studied the effects of PPI in CPG design, and these remain largely unknown [43].

PPI is central to CPG design, and authors recommend that CPG developers use multiple strategies to reach this important goal [43]. Our results suggest that various methods are acceptable and present distinct features that are likely to accommodate different users. Our study also suggests that these methods are feasible for people with TBI. Thus, based on our results, engaging patients experiencing disabilities in CPG co-design appears to be feasible.

However, our pilot study has some limitations. Indeed, we chose a pragmatic approach to evaluate 2 methods of CPG co-design, including a limited number of individuals with TBIs. This practical concern is important in testing the feasibility of the 2 methods in a “real” context of guideline adaptation, but it also raises some issues. To the extent that we had no preliminary data for calculating our sample size, we based our sample on pragmatic criteria. Consequently, our results should be considered with caution as the lack of differences observed between methods may be due to a lack of statistical power. Another limitation of this study is that other relevant data could have been collected to answer our study objective. For example, we did not consider the cost and time required to participate, which could be seen as important information to help decide which method of PPI in CPD co-design to use. We also chose to evaluate 2 methods only on 2 recommendations.

Overall, the results of this pilot study provide initial insight into methods of PPI in CPG co-design. However, further research on these and other methods is crucial to maintain and increase meaningful participation of patients and the public to support quality health care.

## Abbreviations

CPG: clinical practice guideline
PPI: patient and public involvement
PRISMA: preferred reporting items for systematic reviews and meta-analyses
TBI: traumatic brain injury

## References

[1] van de Bovenkamp HM, Zuiderent-Jerak T (2015). An empirical study of patient participation in guideline development: exploring the potential for articulating patient knowledge in evidence-based epistemic settings. Health Expect.

[2] GIN Public Working Group (2015). Patient and Public Involvement in Guidelines. Guidelines International Network.

[3] Graham R, Mancher M (2011). Clinical practice guidelines we can trust. Clinical practice guidelines we can trust.

[4] Armstrong MJ, Mullins CD, Gronseth GS, Gagliardi AR (2017). Recommendations for patient engagement in guideline development panels: A qualitative focus group study of guideline-naïve patients. PLoS ONE.

[5] Légaré F, Boivin A, van der Weijden T, Pakenham C, Burgers J, Légaré J, St-Jacques S, Gagnon S (2011). Patient and Public Involvement in Clinical Practice Guidelines. Med Decis Making.

[6] van de Bovenkamp HM, Trappenburg MJ (2008). Reconsidering Patient Participation in Guideline Development. Health Care Anal.

[7] Kim C, Armstrong MJ, Berta WB, Gagliardi AR (2020). How to identify, incorporate and report patient preferences in clinical guidelines: A scoping review. Health Expect.

[8] Köpke S, Giordano A, Veronese S, Rahn AC, Kleiter I, Basedow‐Rajwich B, Fornari A, Battaglia MA, Drulovic J, Kooij L, Koops J, Mens J, Meza Murillo ER, Milanov I, Milo R, Patti F, Pekmezovic T, Sastre‐Garriga J, Vosburgh J, Voltz R, Bay J, Oliver DJ, Solari A (2018). Patient and caregiver involvement in the formulation of guideline questions: findings from the European Academy of Neurology guideline on palliative care of people with severe multiple sclerosis. Eur J Neurol.

[9] Den Breejen EM, Hilbink MA, Nelen WL, Wiersma TJ, Burgers JS, Kremer JA, Hermens RP (2014). A patient-centered network approach to multidisciplinary-guideline development: a process evaluation. Implementation Sci.

[10] Diaz del Campo P, Gracia J, Blasco JA, Andradas E (2011). A strategy for patient involvement in clinical practice guidelines: methodological approaches. BMJ Quality & Safety.

[11] Armstrong C, Kinnett K, Denger B, Martin A, Coulter I (2019). Participant experiences with a new online modified-Delphi approach for engaging patients and caregivers in developing clinical guidelines. European Journal for Person Centered Healthcare.

[12] Khodyakov D, Grant S, Denger B, Kinnett K, Martin A, Booth M, Armstrong C, Dao E, Chen C, Coulter I, Peay H, Hazlewood G, Street N (2019). Using an Online, Modified Delphi Approach to Engage Patients and Caregivers in Determining the Patient-Centeredness of Duchenne Muscular Dystrophy Care Considerations. Med Decis Making.

[13] Pollard K, Donskoy A, Moule P, Donald C, Lima M, Rice C (2015). Developing and evaluating guidelines for patient and public involvement (PPI) in research. International journal of health care quality assurance.

[14] Armstrong MJ, Gronseth GS, Gagliardi AR, Mullins CD (2020). Participation and consultation engagement strategies have complementary roles: A case study of patient and public involvement in clinical practice guideline development. Health Expect.

[15] Armstrong MJ, Rueda J, Gronseth GS, Mullins CD (2016). Framework for enhancing clinical practice guidelines through continuous patient engagement. Health Expect.

[16] van Wersh A, Eccles M (2001). Involvement of consumers in the development of evidence based clinical guidelines: practical experiences from the North of England evidence based guideline development program. Quality in Health Care.

[17] Diaz del Campo P, Gracia J, Blasco JA, Andradas E (2011). A strategy for patient involvement in clinical practice guidelines: methodological approaches. BMJ Quality & Safety.

[18] Serrano-Aguilar P, del MarTrujillo-Martin MDM, de la Rosa A, Cuellar-Pompa L, Saavedra-Medina H, Linertova R, Perestelo-Perez L, Perez-Ramos J, Rivero-Santana A, Spanish SLE CPG Development Group (2015). Patient participation in a Clinical Guideline Development for Systemic Lupus Erythematosus. Patient Educ Couns.

[19] den Breejen EM, Nelen WL, Knijnenburg JM, Burgers JS, Hermens RP, Kremer JA (2012). Feasibility of a Wiki as a Participatory Tool for Patients in Clinical Guideline Development. J Med Internet Res.

[20] Correa DJ, Kwon C, Connors S, Fureman B, Whittemore V, Jetté N, Mathern GW, Moshé SL, EpiBioS4Rx Public Engagement Core (2019). Applying participatory action research in traumatic brain injury studies to prevent post-traumatic epilepsy. Neurobiol Dis.

[21] Groussard P, Pigot H, Giroux S (2018). From conception to evaluation of mobile services for people with head injury: A participatory design perspective. Neuropsychological Rehabilitation.

[22] Marier-Deschênes P, Gagnon M, Lamontagne M (2019). Co-creation of a post-traumatic brain injury sexuality information toolkit: a patient-oriented project. Disability and Rehabilitation.

[23] Watkins M, Portney L (2009). Foundations of clinical research: applications to practice.

[24] Mills EJ, Chan A, Wu P, Vail A, Guyatt GH, Altman DG (2009). Design, analysis, and presentation of crossover trials. Trials.

[25] 2020. Center for Diseases Control.

[26] Selassie A, Zaloshnja E, Langlois J, Miller T, Jones P, Steiner C (2008). Incidence of long-term disability following traumatic brain injury hospitalization, United States, 2003. J Head Trauma Rehabil.

[27] Zaloshnja E, Miller T, Langlois J, Selassie A (2008). Prevalence of long-term disability from traumatic brain injury in the civilian population of the United States, 2005. J Head Trauma Rehabil.

[28] Lamontagne M, Perreault K, Gagnon M (2014). Evaluation of the acceptability, feasibility and effectiveness of two methods of involving patients with disability in developing clinical guidelines: study protocol of a randomized pragmatic pilot trial. Trials.

[29] Scottish Intercollegiate Guideline Network (2013). Brain injury rehabilitation in adults.

[30] Brouwers MC, Kho ME, Browman GP, Burgers JS, Cluzeau F, Feder G, Fervers B, Graham ID, Grimshaw J, Hanna SE, Littlejohns P, Makarski J, Zitzelsberger L (2010). AGREE II: Advancing guideline development, reporting and evaluation in health care. Journal of Clinical Epidemiology.

[31] Health Council of Canada Understanding Clinical Practice Guidelines: A Video Series primer. Health Council of Canada.

[32] (2017). Random Number Generator 2013. Treck S.

[33] Archambault PM, van de Belt TH, Grajales FJ, Faber MJ, Kuziemsky CE, Gagnon S, Bilodeau A, Rioux S, Nelen WL, Gagnon M, Turgeon AF, Aubin K, Gold I, Poitras J, Eysenbach G, Kremer JA, Légaré F (2013). Wikis and collaborative writing applications in health care: a scoping review. J Med Internet Res.

[34] Archambault PM (2011). WikiBuild: A New Application to Support Patient and Health Care Professional Involvement in the Development of Patient Support Tools. J Med Internet Res.

[35] Souraya S, Carrie JB (2011). Testing the Acceptability and Feasibility of Interventions. Design, Evaluation and Translation of Nursing Intervention. First edition ed.

[36] Pett M (1997). Nonparametric statistics for health care research : statistics for small samples and unusual distributions. Thousand Oaks, Calif.

[37] Hsieh H, Shannon SE (2016). Three Approaches to Qualitative Content Analysis. Qual Health Res.

[38] Graneheim U, Lundman B (2004). Qualitative content analysis in nursing research: concepts, procedures and measures to achieve trustworthiness. Nurse Education Today.

[39] Elo S, Kyngäs H (2008). The qualitative content analysis process. J Adv Nurs.

[40] Shekelle P, Woolf S, Grimshaw JM, Schünemann HJ, Eccles MP (2012). Developing clinical practice guidelines: reviewing, reporting, and publishing guidelines; updating guidelines; and the emerging issues of enhancing guideline implementability and accounting for comorbid conditions in guideline development. Implementation Sci.

[41] Craik C, Glossop J, Sumsion T, Sumsion T (2006). Client-centered practice in occupational therapy.

[42] van der Weijden T, Légaré F, Boivin A, Burgers JS, van Veenendaal H, Stiggelbout AM, Faber M, Elwyn G (2010). How to integrate individual patient values and preferences in clinical practice guidelines? A research protocol. Implementation Sci.

[43] Armstrong MJ, Mullins CD, Gronseth GS, Gagliardi AR (2018). Impact of patient involvement on clinical practice guideline development: a parallel group study. Implementation Sci.

